# *N*-(2,2-Dimethyl­propano­yl)-*N*′-(2-meth­oxy­phen­yl)thio­urea

**DOI:** 10.1107/S1600536812010914

**Published:** 2012-03-21

**Authors:** Maisara A. Kadir, Bohari M. Yamin, M. Sukeri M. Yusof

**Affiliations:** a Department of Chemical Sciences, Faculty of Science and Technology, Universiti Malaysia Terengganu, 21030 Kuala Terengganu, Terengganu, Malaysia; b Department of Chemical Sciences and Food Technology, Faculty of Science and Technology, Universiti Kebangsaan Malaysia, 43650 Bangi, Selangor, Malaysia

## Abstract

In the title compound, C_13_H_18_N_2_O_2_S, the carbonyl­thio­urea fragment is nearly planar with an r.m.s. deviation of 0.0096 Å. The dihedral angle between carbonyl­thio­urea group and the benzene ring is 19.16 (16)°. There are two intra­molecular N—H⋯O hydrogen bonds, which lead to two pseudo-six-membered rings. Weak intra­molecular C—H⋯S hydrogen bonding also occurs.

## Related literature

For related structures, see: Saeed & Flörke (2007 ▶); Yusof *et al.* (2008 ▶); Shoukat *et al.* (2007 ▶). For standard bond lengths, see: Allen *et al.* (1987 ▶).
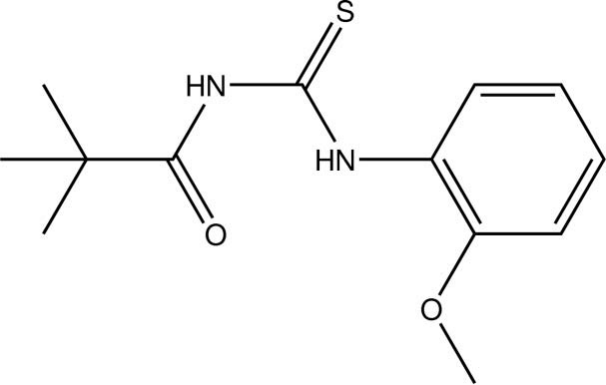


## Experimental

### 

#### Crystal data

C_13_H_18_N_2_O_2_S
*M* *_r_* = 266.35
Orthorhombic, 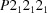

*a* = 5.9181 (10) Å
*b* = 13.492 (2) Å
*c* = 17.592 (3) Å
*V* = 1404.7 (4) Å^3^
*Z* = 4
Mo *K*α radiation
μ = 0.23 mm^−1^
*T* = 273 K
0.50 × 0.14 × 0.09 mm

#### Data collection

Bruker SMART APEX CCD area-detector diffractometer
Absorption correction: multi-scan (*SADABS*; Bruker, 2000 ▶) *T*_min_ = 0.895, *T*_max_ = 0.980
8711 measured reflections
2763 independent reflections
2227 reflections with *I* > 2σ(*I*)
*R*_int_ = 0.045

#### Refinement

*R*[*F* ^2^ > 2σ(*F* ^2^)] = 0.068
*wR*(*F* ^2^) = 0.147
*S* = 1.13
2763 reflections
163 parameters
H-atom parameters constrained
Δρ_max_ = 0.32 e Å^−3^
Δρ_min_ = −0.18 e Å^−3^
Absolute structure: Flack (1983 ▶), 1141
Flack parameter: 0.63 (15)

### 

Data collection: *SMART* (Bruker, 2000 ▶); cell refinement: *SAINT* (Bruker, 2000 ▶); data reduction: *SAINT*; program(s) used to solve structure: *SHELXTL* (Sheldrick, 2008 ▶); program(s) used to refine structure: *SHELXTL*; molecular graphics: *SHELXTL*; software used to prepare material for publication: *SHELXTL* and *PARST* (Nardelli, 1995 ▶).

## Supplementary Material

Crystal structure: contains datablock(s) global, I. DOI: 10.1107/S1600536812010914/bq2346sup1.cif

Structure factors: contains datablock(s) I. DOI: 10.1107/S1600536812010914/bq2346Isup2.hkl

Supplementary material file. DOI: 10.1107/S1600536812010914/bq2346Isup3.cml

Additional supplementary materials:  crystallographic information; 3D view; checkCIF report

## Figures and Tables

**Table 1:** Hydrogen-bond geometry (Å, °)

*D*—H⋯*A*	*D*—H	H⋯*A*	*D*⋯*A*	*D*—H⋯*A*
N2—H2*A*⋯O1	0.86	1.89	2.619 (4)	142
N2—H2*A*⋯O2	0.86	2.17	2.575 (4)	109
C12—H12*A*⋯S1	0.93	2.62	3.235 (4)	124

**Fig. 1.**
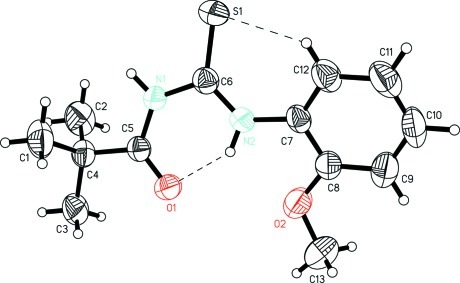
The molecular structure of (I), with displacement ellipsoids drawn at the 50% probability level. Dashed lines show H-bondings.

## supplementary crystallographic information

### Comment

The title compound is analogous to the previously reported, 1-(2-Nitrophenyl)-3-pivaloylthiourea (Saeed and Flörke, 2007) except that the nitro group is replace with methoxy group (Fig. 1). The bond lengths are in normal ranges (Allen *et al.*, 1987) and comparable with other similar molecule reported (Yusof *et al.* 2008; Shoukat *et al.* 2007).

The carbonylthiourea (S1/N1/N2/O1/C4–C7) and phenyl fragments are essentially planar, with rms deviations of 0.0096 Å and 0.0064 Å, respectively. These two fragments inclined at each other at an angle of 19.16(0.16)°. There are two intramolecular hydrogen bonds, N2—H2A···O1 and N2—H2A···O2 leading to two pseudo-six membered rings. Weak C-H···S intramolecular H-bonding is also exist. There is no intermolecular hydrogen bond in the crystal structure.

### Experimental

To a stirring acetone solution (75 ml) of pivaloyl chloride (5.0 g, 0.04 mol) and ammonium thiocyanate (3.15 g, 0.04 mol), 2-methoxyaniline (0.49 g, 0.04 mol) in 40 ml of acetone was added dropwise. The solution mixture was refluxed for 1 h. The resulting solution was poured into a beaker containing some ice blocks. The white precipitate was filtered off and washed with distilled water and cold ethanol before being dried under vacuum. Good quality crystals were obtained by recrystallization from DMF.

### Refinement

H atoms on C were positioned geometrically with C—H 0.93, 0.96 Å, for aromatic and methyl H atoms, respectively, and constrained to ride on their parent atoms with *U*_iso_(H)= *xU*_eq_(C) where *x*=1.5 for methyl H and *x*=1.2 for aromatic H atoms. The H atom attached to oxygen atoms were located from the Fourier difference map and refined isotropically.

### Crystal data

**Table:**

C_13_H_18_N_2_O_2_S	*F*(000) = 568
*M**_r_* = 266.35	*D*_x_ = 1.259 Mg m^−^^3^
Orthorhombic, *P*2_1_2_1_2_1_	Mo *K*α radiation, λ = 0.71073 Å
Hall symbol: P 2ac 2ab	Cell parameters from 770 reflections
*a* = 5.9181 (10) Å	θ = 1.9–26.0°
*b* = 13.492 (2) Å	µ = 0.23 mm^−^^1^
*c* = 17.592 (3) Å	*T* = 273 K
*V* = 1404.7 (4) Å^3^	Slab, colourless
*Z* = 4	0.50 × 0.14 × 0.09 mm

### Data collection

**Table:**

Bruker SMART APEX CCD area-detector diffractometer	2763 independent reflections
Radiation source: fine-focus sealed tube	2227 reflections with *I* > 2σ(*I*)
Graphite monochromator	*R*_int_ = 0.045
Detector resolution: 83.66 pixels mm^-1^	θ_max_ = 26.0°, θ_min_ = 1.9°
ω scan	*h* = −7→6
Absorption correction: multi-scan (*SADABS*; Bruker, 2000)	*k* = −16→16
*T*_min_ = 0.895, *T*_max_ = 0.980	*l* = −20→21
8711 measured reflections	

### Refinement

**Table:**

Refinement on *F*^2^	Secondary atom site location: difference Fourier map
Least-squares matrix: full	Hydrogen site location: inferred from neighbouring sites
*R*[*F*^2^ > 2σ(*F*^2^)] = 0.068	H-atom parameters constrained
*wR*(*F*^2^) = 0.147	*w* = 1/[σ^2^(*F*_o_^2^) + (0.0655*P*)^2^ + 0.2181*P*] where *P* = (*F*_o_^2^ + 2*F*_c_^2^)/3
*S* = 1.13	(Δ/σ)_max_ < 0.001
2763 reflections	Δρ_max_ = 0.32 e Å^−^^3^
163 parameters	Δρ_min_ = −0.18 e Å^−^^3^
0 restraints	Absolute structure: Flack (1983), 1141 Friedel pairs
Primary atom site location: structure-invariant direct methods	Flack parameter: 0.63 (15)

### Special details

**Table:**

Geometry. All e.s.d.'s (except the e.s.d. in the dihedral angle between two l.s. planes) are estimated using the full covariance matrix. The cell e.s.d.'s are taken into account individually in the estimation of e.s.d.'s in distances, angles and torsion angles; correlations between e.s.d.'s in cell parameters are only used when they are defined by crystal symmetry. An approximate (isotropic) treatment of cell e.s.d.'s is used for estimating e.s.d.'s involving l.s. planes.
Refinement. Refinement of *F*^2^ against ALL reflections. The weighted *R*-factor *wR* and goodness of fit *S* are based on *F*^2^, conventional *R*-factors *R* are based on *F*, with *F* set to zero for negative *F*^2^. The threshold expression of *F*^2^ > σ(*F*^2^) is used only for calculating *R*-factors(gt) *etc*. and is not relevant to the choice of reflections for refinement. *R*-factors based on *F*^2^ are statistically about twice as large as those based on *F*, and *R*- factors based on ALL data will be even larger.

### Fractional atomic coordinates and isotropic or equivalent isotropic displacement parameters (Å^2^)

**Table:**

	*x*	*y*	*z*	*U*_iso_*/*U*_eq_	
S1	0.1312 (2)	0.79180 (7)	0.57763 (7)	0.0693 (4)	
O1	0.3640 (5)	0.47260 (17)	0.56305 (13)	0.0559 (7)	
O2	0.7537 (5)	0.5571 (2)	0.67865 (15)	0.0662 (8)	
C6	0.2391 (6)	0.6781 (2)	0.58348 (19)	0.0442 (8)	
N2	0.3935 (5)	0.6446 (2)	0.63138 (15)	0.0453 (7)	
H2A	0.4306	0.5836	0.6243	0.054*	
N1	0.1602 (5)	0.60835 (18)	0.53095 (15)	0.0427 (7)	
H1A	0.0573	0.6290	0.5003	0.051*	
C5	0.2250 (6)	0.5112 (2)	0.52178 (18)	0.0384 (8)	
C8	0.7025 (7)	0.6429 (3)	0.71748 (19)	0.0474 (9)	
C9	0.8272 (8)	0.6805 (3)	0.7766 (2)	0.0623 (11)	
H9A	0.9588	0.6489	0.7924	0.075*	
C4	0.1096 (6)	0.4544 (2)	0.45732 (18)	0.0445 (8)	
C7	0.5083 (7)	0.6910 (2)	0.69201 (17)	0.0434 (9)	
C3	0.2134 (8)	0.3508 (3)	0.4531 (2)	0.0682 (12)	
H3A	0.1901	0.3171	0.5005	0.102*	
H3B	0.3724	0.3562	0.4432	0.102*	
H3C	0.1427	0.3140	0.4128	0.102*	
C2	0.1488 (9)	0.5089 (3)	0.3823 (2)	0.0726 (13)	
H2B	0.0831	0.5738	0.3850	0.109*	
H2C	0.0798	0.4724	0.3416	0.109*	
H2D	0.3082	0.5146	0.3731	0.109*	
C12	0.4381 (8)	0.7757 (3)	0.7293 (2)	0.0599 (12)	
H12A	0.3061	0.8075	0.7142	0.072*	
C11	0.5629 (10)	0.8133 (3)	0.7888 (2)	0.0731 (15)	
H11A	0.5165	0.8711	0.8129	0.088*	
C10	0.7544 (9)	0.7660 (3)	0.8125 (2)	0.0714 (14)	
H10A	0.8365	0.7915	0.8532	0.086*	
C13	0.9241 (8)	0.4949 (3)	0.7089 (2)	0.0744 (13)	
H13A	0.9422	0.4379	0.6769	0.112*	
H13B	0.8817	0.4740	0.7591	0.112*	
H13C	1.0642	0.5307	0.7113	0.112*	
C1	−0.1417 (7)	0.4454 (3)	0.4742 (3)	0.0734 (12)	
H1B	−0.2072	0.5103	0.4777	0.110*	
H1C	−0.1626	0.4110	0.5215	0.110*	
H1D	−0.2141	0.4090	0.4341	0.110*	

### Atomic displacement parameters (Å^2^)

**Table:**

	*U*^11^	*U*^22^	*U*^33^	*U*^12^	*U*^13^	*U*^23^
S1	0.0848 (8)	0.0424 (5)	0.0806 (7)	0.0145 (6)	−0.0263 (7)	−0.0102 (5)
O1	0.0650 (17)	0.0465 (13)	0.0562 (14)	0.0105 (13)	−0.0175 (14)	−0.0061 (11)
O2	0.068 (2)	0.0701 (18)	0.0609 (15)	0.0127 (16)	−0.0192 (15)	−0.0080 (14)
C6	0.045 (2)	0.0429 (18)	0.0448 (18)	−0.0036 (16)	0.0009 (18)	0.0016 (15)
N2	0.0535 (19)	0.0360 (14)	0.0465 (16)	0.0025 (14)	−0.0089 (15)	−0.0053 (12)
N1	0.0445 (17)	0.0396 (15)	0.0440 (15)	0.0033 (13)	−0.0109 (14)	−0.0057 (12)
C5	0.0366 (19)	0.0403 (17)	0.0385 (17)	−0.0044 (15)	0.0033 (15)	0.0015 (14)
C8	0.054 (2)	0.048 (2)	0.0405 (18)	−0.0111 (18)	−0.0028 (17)	0.0069 (16)
C9	0.061 (3)	0.074 (3)	0.051 (2)	−0.020 (2)	−0.010 (2)	0.008 (2)
C4	0.039 (2)	0.0484 (19)	0.0462 (19)	−0.0020 (17)	0.0003 (16)	−0.0068 (15)
C7	0.057 (2)	0.0386 (18)	0.0351 (18)	−0.0119 (18)	−0.0021 (17)	0.0025 (14)
C3	0.074 (3)	0.057 (2)	0.074 (3)	0.002 (2)	−0.016 (2)	−0.027 (2)
C2	0.100 (4)	0.078 (3)	0.039 (2)	−0.011 (3)	−0.004 (2)	−0.0070 (19)
C12	0.085 (3)	0.050 (2)	0.045 (2)	−0.007 (2)	−0.003 (2)	−0.0055 (17)
C11	0.119 (5)	0.050 (2)	0.050 (2)	−0.015 (3)	−0.002 (3)	−0.0072 (18)
C10	0.100 (4)	0.068 (3)	0.046 (2)	−0.041 (3)	−0.016 (3)	0.000 (2)
C13	0.063 (3)	0.088 (3)	0.073 (3)	0.012 (2)	−0.007 (2)	0.008 (2)
C1	0.051 (3)	0.080 (3)	0.090 (3)	−0.014 (2)	−0.002 (2)	−0.021 (2)

### Geometric parameters (Å, º)

**Table:**

S1—C6	1.665 (3)	C7—C12	1.382 (5)
O1—C5	1.215 (4)	C3—H3A	0.9600
O2—C8	1.378 (5)	C3—H3B	0.9600
O2—C13	1.416 (5)	C3—H3C	0.9600
C6—N2	1.323 (4)	C2—H2B	0.9600
C6—N1	1.399 (4)	C2—H2C	0.9600
N2—C7	1.411 (4)	C2—H2D	0.9600
N2—H2A	0.8600	C12—C11	1.378 (6)
N1—C5	1.375 (4)	C12—H12A	0.9300
N1—H1A	0.8600	C11—C10	1.366 (7)
C5—C4	1.530 (5)	C11—H11A	0.9300
C8—C9	1.373 (5)	C10—H10A	0.9300
C8—C7	1.394 (5)	C13—H13A	0.9600
C9—C10	1.385 (6)	C13—H13B	0.9600
C9—H9A	0.9300	C13—H13C	0.9600
C4—C1	1.522 (5)	C1—H1B	0.9600
C4—C2	1.529 (5)	C1—H1C	0.9600
C4—C3	1.529 (5)	C1—H1D	0.9600
			
C8—O2—C13	117.9 (3)	C4—C3—H3C	109.5
N2—C6—N1	114.9 (3)	H3A—C3—H3C	109.5
N2—C6—S1	128.3 (3)	H3B—C3—H3C	109.5
N1—C6—S1	116.8 (3)	C4—C2—H2B	109.5
C6—N2—C7	131.5 (3)	C4—C2—H2C	109.5
C6—N2—H2A	114.3	H2B—C2—H2C	109.5
C7—N2—H2A	114.3	C4—C2—H2D	109.5
C5—N1—C6	128.7 (3)	H2B—C2—H2D	109.5
C5—N1—H1A	115.7	H2C—C2—H2D	109.5
C6—N1—H1A	115.7	C11—C12—C7	120.2 (4)
O1—C5—N1	121.9 (3)	C11—C12—H12A	119.9
O1—C5—C4	122.0 (3)	C7—C12—H12A	119.9
N1—C5—C4	116.1 (3)	C10—C11—C12	120.4 (4)
C9—C8—O2	124.6 (4)	C10—C11—H11A	119.8
C9—C8—C7	121.0 (4)	C12—C11—H11A	119.8
O2—C8—C7	114.4 (3)	C11—C10—C9	120.5 (4)
C8—C9—C10	119.1 (4)	C11—C10—H10A	119.7
C8—C9—H9A	120.5	C9—C10—H10A	119.7
C10—C9—H9A	120.5	O2—C13—H13A	109.5
C1—C4—C2	110.8 (4)	O2—C13—H13B	109.5
C1—C4—C3	109.2 (3)	H13A—C13—H13B	109.5
C2—C4—C3	109.6 (3)	O2—C13—H13C	109.5
C1—C4—C5	109.4 (3)	H13A—C13—H13C	109.5
C2—C4—C5	109.4 (3)	H13B—C13—H13C	109.5
C3—C4—C5	108.4 (3)	C4—C1—H1B	109.5
C12—C7—C8	118.7 (3)	C4—C1—H1C	109.5
C12—C7—N2	125.5 (4)	H1B—C1—H1C	109.5
C8—C7—N2	115.7 (3)	C4—C1—H1D	109.5
C4—C3—H3A	109.5	H1B—C1—H1D	109.5
C4—C3—H3B	109.5	H1C—C1—H1D	109.5
H3A—C3—H3B	109.5		
			
N1—C6—N2—C7	−178.8 (3)	O1—C5—C4—C3	−4.2 (5)
S1—C6—N2—C7	2.0 (6)	N1—C5—C4—C3	176.6 (3)
N2—C6—N1—C5	−1.5 (5)	C9—C8—C7—C12	−2.3 (5)
S1—C6—N1—C5	177.9 (3)	O2—C8—C7—C12	177.6 (3)
C6—N1—C5—O1	2.2 (5)	C9—C8—C7—N2	−179.4 (3)
C6—N1—C5—C4	−178.6 (3)	O2—C8—C7—N2	0.5 (4)
C13—O2—C8—C9	10.6 (5)	C6—N2—C7—C12	19.9 (6)
C13—O2—C8—C7	−169.3 (3)	C6—N2—C7—C8	−163.3 (3)
O2—C8—C9—C10	−178.1 (3)	C8—C7—C12—C11	2.2 (6)
C7—C8—C9—C10	1.7 (5)	N2—C7—C12—C11	178.9 (3)
O1—C5—C4—C1	114.7 (4)	C7—C12—C11—C10	−1.4 (6)
N1—C5—C4—C1	−64.5 (4)	C12—C11—C10—C9	0.8 (6)
O1—C5—C4—C2	−123.7 (4)	C8—C9—C10—C11	−1.0 (6)
N1—C5—C4—C2	57.1 (4)		

### Hydrogen-bond geometry (Å, º)

**Table:**

*D*—H···*A*	*D*—H	H···*A*	*D*···*A*	*D*—H···*A*
N2—H2*A*···O1	0.86	1.89	2.619 (4)	142
N2—H2*A*···O2	0.86	2.17	2.575 (4)	109
C12—H12*A*···S1	0.93	2.62	3.235 (4)	124
